# Doping May Be Responsible for*De Novo* Mitochondrial Disorder

**DOI:** 10.15171/apb.2019.021

**Published:** 2019-06-01

**Authors:** Josef Finsterer

**Affiliations:** Krankenanstalt Rudolfstiftung, Messerli Institute, Wien, Austria.

## Dear Editor,

In a recent article by Jackson et al, a 9 years-old girl with microcephaly, developmental delay, intellectual disability, and ataxia was presented.^1^ Already in early infancy the patient developed sucking weakness, motor development was delayed (sitting independently at age 15m, walking independently at age 22m), and she showed a tendency to freeze easily.^1^ The patient had gallstones at age 4m. From age 7m she developed ataxia, microcephaly, short stature, with a delay in motor development, and serum and CSF lactate were repeatedly elevated. Biochemical investigations of primary fibroblast cultures and muscle homogenate showed severe reduction of complex-V of the respiratory chain with a residual activity of 25% in muscle and 28% in fibroblasts.^1^ Ultrastructurally, mitochondria were distorted with aberrant cristae formation. Western blot analysis of mitochondrial proteins showed reduced complex-V amount and impaired complex-V assembly.^1^ Sequencing of the mtDNA-located genes *MT-ATP6/8* revealed the heteroplasmic insertion C at position 8611 (m.8611_8612insC).^1^

Since the mother of the index case was a top endurance athlete in the former German Democratic Republic (GDR).^1^ since systematic doping with androgenic-anabolic steroids (AAS) occurred in the GDR,^2^ since AAS are cytotoxic and mutagenic in mice,^3^ since AAS may induce mtDNA mutations by increasing oxidative stress,^4^ and since the *ATP6* mutation in the index case was *de novo*, we strongly assume that the mother of the index case had undergone systematic doping with AAS during her active career as an athlete as well and that chronic misuse of AAS induced mtDNA mutations in her oocytes.

Generally, reported side effects of AAS doping include hyperandrogenism, adrenal hyperplasia, polycystic ovary syndrome, brain-connectivity disorder, exaggerated self-confidence, reckless behaviour, aggressiveness, psychotic symptoms, arrhythmias, cardiomyopathies, sudden death. increased hematocrit, coagulopathy (thromboembolism, intracardiac thrombosis, stroke), hepatopathy, cholestasis, peliosis, hyperbilirubinemia with cholemic nephrosis, kidney failure, adenomas, carcinomas, and myopathy. AAS withdrawal may be accompanied by depression and suicidal intentions.

Unfortunately, the issue of previous doping in this particular mother is not discussed in the article by Jackson et al.^1^ It is not addressed if the mother developed any of the potential side-effects of AAS during her active time as an athlete or later. The authors do not mention the interval between the end of the presumed doping and the birth of her daughter. It is not mentioned if this mother gave birth to other children as well and if they manifested with the mitochondrial disorder as well.

Furthermore, the index case was not prospectively investigated for manifestations other than the ones reported but previously described in association with *ATP6* mutations. This is of relevance, since *ATP6* mutations cause multisystem disease manifesting in the central nervous system (CNS, ataxia, seizures, bilateral striatal necrosis, developmental delay, mental retardation, Leigh-syndrome, optic neuropathy, spastic paraplegia), eyes (pigmentary retinopathy, astigmatism, exophoria), heart (cardiomyopathy), muscles (myopathy), peripheral nerves (polyneuropathy, thermosensitivity), blood (sideroblastic anemia), and others (lactic acidosis, malignancy). Since the CNS is predominantly affected in *ATP6* mutations, there is a need that cerebral MRI and electroencephalography are repeatedly carried out during follow-up. Normal cerebral MRI at age 2y does not exclude that it becomes abnormal during follow-up. Absence of cardiac symptoms does not exclude that the heart is subclinically affected or becomes affected during follow-up.

The interesting report could be more meaningful if the mother’s history as an athlete would have been explored more extensively and if additional information about prospective and recurrent follow-up investigations in the index case were provided.

## Ethical Issues

Not applicable.

## Conflict of Interest

There is no conflict of interest to declare.

## References

[1] Jackson CB, Hahn D, Schroter B, Richter U, Battersby BJ, Schmitt-Mechelke T (2017). A novel mitochondrial ATP6 frameshift mutation causing isolated complex V deficiency, ataxia and encephalomyopathy. Eur J Med Genet.

[2] Fitch KD (2008). Androgenic-anabolic steroids and the Olympic Games. Asian J Androl.

[3] Meireles JR, Oliveira SV, Costa-Neto AO, Cerqueira EM (2013). Genotoxic and cytotoxic effects of testosterone cypionate (deposteron((R))). Mutat Res.

[4] Santos MA, Oliveira CV, Silva AS (2014). Adverse cardiovascular effects from the use of anabolic-androgenic steroids as ergogenic resources. Subst Use Misuse.

